# Patients with severe COVID‐19 have reduced circulating levels of angiotensin‐(1–7): A cohort study

**DOI:** 10.1002/hsr2.564

**Published:** 2022-03-14

**Authors:** Seyed Mohammad Seyedmehdi, Fatemeh Imanparast, Pegah Mohaghegh, Saeed Mahmoudian, Mona Karimi Dehlaqi, Fatemeh Mehvari, Mihan Pour Abdullah

**Affiliations:** ^1^ Dr. Masih Daneshvari Hospital Shahid Beheshti University of Medical Sciences Tehran Iran; ^2^ Infectious Diseases Research Center (IDRC) Arak University of Medical Sciences Arak Iran; ^3^ Department of Biochemistry and Genetics, Faculty of Medicine Arak University of Medical Sciences Arak Iran; ^4^ Community and Preventive Medicine Specialist, Department of Community Medicine, Faculty of Medicine Arak University of Medical sciences Arak Iran

**Keywords:** angiotensin 1–7, angiotensin‐converting enzyme 2, comorbidity, coronavirus disease 19, severe acute respiratory syndrome coronavirus 2

## Abstract

**Background and Aims:**

Angiotensin‐converting enzyme 2 (ACE2) acts as a functional receptor for the entry of severe acute respiratory syndrome coronavirus 2 into host cells. Angiotensin (1–7) (Ang (1–7)) obtained from the function of ACE2 improves heart and lung function. We investigated the relationship between Ang (1–7) level and disease severity in patients with coronavirus disease 2019 (COVID‐19).

**Methods:**

This cohort study was carried out at Masih Daneshvari Hospital in Tehran, Iran from September 2020 to October 2020. To do so, the Ang (1–7) levels of 331 hospitalized COVID‐19 patients with and without underlying disease were measured by ELISA kit. The need for oxygen, intubation, and mechanical ventilation were recorded for all the patients.

**Results:**

Results showed a significant inverse relationship between the levels of Ang 1–7 and the severity of the disease (needed oxygen, intubation, and mechanical ventilation). According to the results, median (interquartile range) of Ang (1–7) levels was significantly lower in patients who needed oxygen versus those who needed no oxygen (44.50 (91) vs. 82.25 (68), *p* = 0.002), patients who needed intubation and mechanical ventilation versus those who did not (9.80 (62) vs. 68.70 (102), *p* < 0.000) and patients hospitalized in an intensive care unit (ICU) than people hospitalized in other wards. We also found that the older patients were more in need of ICU and mechanical ventilation than younger patients.

**Conclusions:**

Higher levels of Ang (1–7) have been associated with decreased disease severity. Besides this, we perceived that synthetic Ang 1–7 peptides may be useful to treat and reduce the complications of COVID‐19.

## 1 INTRODUCTION

As a newfound illness, coronavirus disease 2019 (COVID‐19) pneumonia is rapidly spreading around the world. The renin–angiotensin–aldosterone system plays a critical role in COVID‐ 19 pathogenesis.

Recent studies have shown a crucial role of angiotensin‐converting enzyme 2 (ACE2) in the pathogenesis of severe acute respiratory syndrome coronavirus 2 (SARS‐CoV‐2) infection, which causes COVID‐19. ACE2 acts as a functional receptor for the entry of SARS‐CoV‐2 into host cells. ACE2 as a membrane‐bound aminopeptidase converts angiotensin I (Ang I) and Ang II into Ang (1–9) and (Ang 1–7), respectively, and is expressed in various human organs.^1^ Ang (1–7) exerts vasodilation, vascular protection, anti‐fibrosis, anti‐proliferation, and anti‐inflammation actions. The heart, brain, and kidney are major sources to produce Ang (1–7). This peptide as a vasodilator agent plays an important role in improving heart and lung functions.^2^

On the one hand, ACE2 is overexpressed in heart failure, arterial hypertension, and diabetes mellitus. So, people with these underlying diseases are more likely to get infected by COVID‐19. On the other hand, SARS‐CoV‐2 downregulates ACE2 and reduces Ang (1–7) after infection. As a result, the high severity and high mortality rate among these patients could be due to reasons, such as vasoconstriction, vascular damage, fibrosis, proliferation, and inflammation due to reduction of Ang (1–7).^3^

Given the key role of ACE2 in infection of the virus and the protective roles of Ang (1–7) as its product, we hypothesized that there was an indirect relationship between Ang (1–7) level and disease severity in patients with COVID‐19.

However, no reports are available regarding Ang (1–7) levels in the COVID‐19 patients with various disease severities. Therefore, in this study, we evaluated the relationship between Ang (1–7) levels and disease severity in the COVID‐19 patients with and without the underlying diseases, such as cardiovascular diseases and diabetes.

## 2 MATERIALS AND METHODS

This cohort study was carried out at Masih Daneshvari Hospital in Tehran, Iran from September 1, 2020 to October 30, 2020. In this study, the Ang (1–7) levels in patients with the COVID‐19 disease with different severity were measured and compared. We calculated the sample size according to the following formula:

$$N=\frac{{\left(z_{1-\frac{\alpha }{2}}\right)}^{2}\text{pq}}{d^{2}},$$

where expected range (*α*) = 0.05, *z* score (*z*) = 1.96, and expected range (*d*) =  0.93, and (*q*) = 1 − *p*, where *p* is the expected prevalence of intensive care unit (ICU) admission in COVID‐19 patients according to the study of Mehta et al.^4^ We reached the sample size of 343 people with the COVID‐ 19 disease.

To decrease the selection bias, due to the specific conditions of this emerging disease and the existence of little information about this disease, we considered all the people who were referred to the hospital and were hospitalized for this disease for a period of time (from September 1, 2020 to October 30, 2020) as the prototype sample size. About 1853 people with the COVID‐19 disease were hospitalized during this period. For all patients, questionnaires were completed by nurses. The questionnaire contained all inclusion and exclusion criteria except noticeably changed in lifestyle in terms of nutrition, especially the use of antioxidants and vitamin D and physical activity during the 6 months before this disease. The blood samples were taken from all patients after admission before starting any additional treatment (medication, oxygen therapy, etc.) to evaluate clinical trials. For all patients, excess serums (20 min; 3000 rpm) were stored at −80°C for further research on this emerging disease. The patients' clinical symptoms, including ICU admission, the use of mechanical ventilation, oxygen need, fever, and gastrointestinal disorders were drawn out from the patient's medical record after being discharged from the hospital. At the end of the study period, after reviewing the questionnaires, 645 patients were selected based on the inclusion and exclusion criteria in this study. Of these patients, those who had significantly changed their diet or lifestyle during the 6 months before illness or were not satisfied with participating in the study were excluded, and the remaining 331 patients were considered as the final sample volume for Ang (1–7) level assay.

Inclusion criteria were infection with COVID‐19 approved by real‐time PCR test and hospitalization, while exclusion criteria included patients with COVID‐19 being reluctant to participate in the study, with several underlying diseases simultaneously, have died during this period, smokers, and drug users, as well as patients who had noticeably changed their lifestyle in terms of nutrition, especially the use of antioxidants and vitamin D and physical activity during the 6 months before the disease.

### 2.1 Biochemical assessments and clinical symptoms

The blood samples of all the subjects were first taken immediately after the patients were admitted to the hospital when it was confirmed that the patient had COVID‐19. Next, aliquot samples of serums were saved following centrifugation (20 min; 3000 rpm) at −80°C. Then, the samples' levels of Ang (1–7) were measured by ELISA kit (Germany, Zellbio), on the basis of the biotin double antibody sandwich technology, with assay range 40–1280 ng/L, sensitivity 5 ng/L, intra‐assay (coefficient of variation [CV] < 10%), and interassay (CV < 12%).

Finally, the patients' clinical symptoms, including ICU admission, the use of mechanical ventilation, oxygen need, fever, and gastrointestinal disorders were drawn out from the patient's medical record after being discharged from the hospital

### 2.2 Statistical analysis

Statistical analyses were performed using the IBM Statistical Package for Social Sciences (SPSS, version 17; SPSS Inc.). Continuous quantitative variables were reported as medians and interquartile ranges (IQRs) if they had nonparametric distribution and categorical variables were described as numbers (percentages). Kolmogorov–Smirnov test was used to assess normality of Ang (1–7). The median and IQR have been used to describe the Ang (1–7). Categorical variables were compared with the use of the *χ* ^2^ test, while continuous variables were compared with the Mann–Whitney *U* test. A *χ* ^2^ test was performed to examine the correlation between fever, gastrointestinal symptoms, an underlying disease with ICU admission, and oxygen need. Mann–Whitney *U* test was performed to compare Ang (1–7) levels according to severity outcome (ICU admission and Oxygen need) with 95% confidence intervals (CIs). All the significance tests were two‐tailed. The significance level of the *p* value was set at a  <0.05.

## 3 RESULTS

This cohort study was carried out from September 2020 to October 2020 Three hundred and thirty‐one patients with COVID‐19 participated in this study.

According to the results of this study, The mean ± SD age of patients was 57.69 ± 15.21 years. The mean ± SD of body mass index (BMI) of patients was 28.23 ± 10.99 kg/m^2^ (Table 1). The amount of Ang (1–7) levels in different groups of age, sex, and BMI in this study were not different.

**Table 1:** General and clinical characteristics of the samples

Variables	Category		Frequency (%)
General characteristics of the sample			
Age		<30	9 (2.7)
		30–59	175 (52.9)
		≥60	147 (44.4)
BMI		<18.5 (underweight)	4 (1.2)
		18.5–25 (normal)	87 (26.3)
		25–29.9 (overweight)	150 (45.3)
		≥30 (obesity)	83 (25.1)
Sex		Female	139 (42)
		Male	192 (58)
Clinical characteristics of the sample			
Fever	<37		312 (94.3)
	≥37		19 (5.7)
Gastrointestinal symptoms	Yes		7 (2.1)
Underlying disease	Yes		204 (61.6)
Cardiovascular disease	Yes		136 (49.8)
Respiratory diseases	Yes		49 (27.9)
Kidney diseases	Yes		24 (15.9)
Diabetic diseases	Yes		86 (40.4)

Abbreviation: BMI, body mass index.

Table 2 showed the clinical implications (ICU admission, oxygen, and incubation need) in different groups of age, BMI, sex, and underlying diseases (cardiovascular, respiratory, kidney, diabetic, and thyroid diseases). Patients with ≥60 years had significantly more clinical complications like the need for an ICU and ventilator compared to younger ages. There were no significant differences in terms of the clinical complications between different BMI and/or sexes. While the clinical complications as the need for an ICU and ventilator are significantly higher in patients with underlying diseases, especially this difference is in patients with diabetes than nondiabetics.

**Table 2:** Relation of clinical implications (ICU admission, oxygen need, and incubation) and underlying diseases (cardiovascular, respiratory, kidney, diabetic, and thyroid diseases)

Variables	Category frequency (%)	ICU admission and incubation frequency (%)		*χ* ^2^ statistic	*p* Value*	Oxygen need frequency (%)		*χ* ^2^ statistic	*p* Value*
		Yes	No			Yes	No		
Fever	<37	71 (88.8)	241 (96)	5.91	0.015	244 (95.7)	68 (89.5)	4.17	0.04
	≥37	9 (11.3)	10 (4)			11 (4.3)	8 (10.5)		
Gastrointestinal symptoms	No	75 (93.8)	249 (99.6)	11.62	0.001	250 (98.4)	74 (97.4)	0.37	0.54
	Yes	5 (6.3)	1 (0.4)			4 (1.6)	2 (2.6)		
Underlying disease	No	22 (27.5)	105 (41.8)	5.27	0.02	93 (36.5)	34 (44.7)	1.69	0.19
	Yes	58 (72.5)	146 (58.2)			162 (63.5)	42 (55.3)		
Cardiovascular disease	No	38 (45.5)	157 (62.5)	5.67	0.02	147 (57.6)	48 (63.2)	0.73	0.39
	Yes	42 (52.5)	94 (37.5)			108 (42.4)	28 (36.8)		
Respiratory diseases	No	73 (91.3)	209 (83.3)	3.06	0.08	218 (85.5)	64 (84.2)	0.76	0.78
	Yes	7 (8.8)	42 (16.7)			37 (14.5)	12 (15.8)		
Kidney diseases	No	73 (91.3)	234 (93.2)	0.353	0.55	233 (91.4)	74 (97.4)	3.13	0.07
	Yes	7 (8.8)	17 (6.8)			22 (8.6)	2 (2.6)		
Diabetic patients	No	48 (60)	197 (78.5)	10.78	0.001	187 (73.3)	58 (76.3)	0.27	0.60
	Yes	32 (40)	54 (21.5)			68 (26.7)	18 (23.7)		
Thyroid disorders	No	79 (98.8)	239 (95.2)	2.00	0.16	245 (96.1)	73 (96.1)	0.00	>0.99
	Yes	1 (1.3)	12 (4.8)			10 (3.9)	3 (3.9)		

Abbreviation:  ICU, intensive care unit.

* *χ* ^2^ test.

Table 3 showed the circulating levels of Ang (1–7) according to the disease severity. There was a statistically significant association between the median (IQR) of Ang (1–7) levels of patients with the disease severity. It means that the hospitalized COVID‐19 patients who needed ICU and intubation had lower levels of Ang (1–7) than those who did not (9.80 (62) vs. 68.70 (102) pg/ml, *p* < 0.001), and patients who needed oxygen and those who needed no oxygen (44.50 (91) vs. 82.25 (68) pg/ml, *p* value = 0.002). Finally, there is a significant indirect relationship between the severity of the disease and Ang (1‐7) levels.

**Table 3:** Circulating levels of Ang‐(1–7) according to disease severity

Variables	Category	Frequency (%)	Ang‐(1–7) level, median (IQR)	CI	*p* Value*
Oxygen need	Yes	255 (77)	44.50 (91)	48.62–63.17	0.002
	No	76 (23)	82.25 (68)	65.53–88.48	
ICU admission and incubation	Yes	80 (24.2)	9.80 (62)	26.42–48.55	<0.001
	No	251 (75.8)	68.70 (102)	60.92–75.39	
	Yes	13 (32.5)	32.20		0.818

Abbreviations: Ang, angiotensin; CI, confidence interval; IQR, interquartile range.

* Mann–Whitney *U* test and Kruskal–Wallis test.

## 4 DISCUSSION

In this study, we evaluated the relationship between Ang (1–7) level with disease severity in COVID‐19 patients hospitalized in Hospital. Also, we studied the association of underlying diseases and disease severity in COVID‐19 patients. We observed the disease was more severe in COVID‐19 patients with underlying diseases, such as cardiovascular, respiratory, and diabetes than patients without such underlying diseases. Also, Ang 1–7 levels in both groups of patients with and without underlying diseases hospitalized in ICU were lower than patients hospitalized in other wards. In agreement with the results of our study, Huang et al.^5^ reported that COVID‐19 patients with underlying diseases, such as hypertension, cardiovascular, and diabetes diseases showed more severe disease severity such as ICU admission. They also showed that the plasma levels of inflammatory factors such as interleukin‐2 (IL‐2), IL‐7, IL‐10, and tumor necrosis factor‐α were higher in ICU patients.^5^ SARS‐CoV‐2 enters host cells via ACE2, which is expressed in various human organs and its upregulation increases the susceptibility of individuals to this infection.^6^ Following the binding of SARS‐CoV‐2 to ACE2 as the receptor and SARS‐CoV‐2/ACE2 internalization, Ang II and Ang‐(1–7) level increases and decrease, respectively.^7^ But the report of Valle Martins, contrary to our findings and the previous hypotheses, reported that the amount of Ang‐(1–7) increased in COVID‐19 patients. One of the reasons for this difference is that in our study we compared the amount of this peptide in COVID‐19 patients with different severity, but in their study, they compared the level of peptide in COVID‐19 patients with non‐COVID‐19 volunteers. According to the results of our study and theirs, it is possible that at the beginning of the disease, levels of ACE2 and Ang (1–7) increase due to stimulation by the virus, Then, as the disease progresses and the virus load increases, Ang (1–7) decreases due to more AEC2 internalization. There is an indirect relationship between virus load and ACE2 levels. Studies have shown the severity of the disease in patients with the underlying disease is higher than in healthy patients due to the high expression of ACE2 and inflammatory factors.^8^ On the other hand, Ang II is responsible for increasing blood pressure in cardiovascular and diabetic diseases.^9^ There are controversial and contradictory views on the benefits and harms of the medications that upregulate and/or downregulate ACE2 levels to control COVID 19.^10^, ^11^ The point to consider is that, similar to its overexpression, ACE2 blockade has a role in the pathogenesis of SARS‐CoV‐2 by increasing Ang II activity with simultaneous barricading the functions of Ang (1–7)/MasR axis. Disturbing the balance between Ang II and Ang (1–7) activities could also cause acute respiratory distress syndrome (ARDS) due to interstitial pulmonary fibrosis, cardiomyopathy, and shock reported in COVID‐19 patients.^12^ While Guo et al.^13^ reported mortality in 36.8% of patients on ACE inhibitors/ARBs versus in 25.6% individuals without their use. One possible reason for Guo's unexpected results could be the ACE strengthening effect on the immune system and promoting tissue remodeling through the formation of reactive oxygen species (ROS).^14^ In a pilot clinical trial, Khan et al.^15^ reported the reducing and increasing effects of recombinant human ACE2 on Ang II levels and Ang (1–7) levels, respectively in ARDS. Santuchi et al.^16^ reported the anti‐inflammatory properties of Ang (1–7). Further, Yu et al.^17^ showed that Ang (1–7) could offer anti‐inflammatory properties in pancreatitis by inhibiting the p38 MAPK/NF‐κB signaling pathway. Likewise, some other studies have shown the anti‐inflammatory and anti‐fibrotic effects, increasing insulin secretion, and reducing ROSs of Ang (1–7).^18^, ^19^, ^20^ In another clinical trial, Rodgers et al.^21^ reported that Ang (1–7) is safe in human use.

As far as we know, no study has been so far conducted regarding Ang (1–7) level in COVID‐19 patients. In this study, levels of this peptide were found to be lower in patients with higher severity, although the severity of the disease was higher in the elderly, the levels of this peptide did not differ at different ages. Despite the fact that no age‐dependent ACE2 levels have been observed in human studies, one of the reasons for the severity of this disease in elderly patients could be higher Ang II and lower MasR expression (as the putative receptor for Ang1–7) in older patients compared to younger ones.^22^, ^23^ MasR agonists have protective effects in COVID‐19 patients.^24^ Monteil^25^ reported that soluble ACE2 might block SARS‐CoV‐2 infections in the early stages, but it has no efficacy when the COVID19 disease becomes more severe.

In this study, moreover, disease severity and Ang 1–7 levels were not different between male and female patients. In an investigation on 99 patients infected with SARS‐CoV‐2, Chen et al.^26^ showed that males were more susceptible to infection than females because ACE2‐expressing lung cells were more abundant in males. Li et al.^27^ also indicated that SARS‐CoV‐2 may equally infect individuals of different sexes, ages, and races. They further pointed out that the disease severity in people of different ages and gender depends on the host immune response to SARS‐CoV‐2 infection.

One of the limitations of this study was the nonparticipation of non‐COVID‐19 volunteers and COVID‐19 patients who did not need to be hospitalized, as well as those who unfortunately died of this disease. The other limitation was the lack of peptide assay by gold‐standard methods such as liquid chromatography‐tandem mass spectrometry. The lack of matching of factors such as age, sex, and BMI was another limitation of this study; however, the amount of Ang (1–7) in different groups of age, sex, and BMI in this study was not different.

## 5 CONCLUSION

In the current research, we firstly found that there is a significant and indirect relationship between Ang (1–7) levels and the severity of COVID‐19 disease, both in patients with and without underlying diseases. Therefore, further studies, especially randomized controlled clinical trials are needed to clearly delineate the benefits of Ang (1–7) actions in COVID 19 patients.

## CONFLICTS OF INTEREST

The authors declare no conflicts of interest.

## ETHICS STATEMENT

This study was approved by the human research ethics committee at Arak University of Medical Sciences, Arak, Iran (IR. ARAKMU. REC.1399.3691) and the human research ethics committee at Shahid Beheshti University of Medical Sciences, Tehran, Iran (IR. SBMU. NRITLD. REC.1399.153). We confirm that consent was obtained from all patients before participating in this study.

## AUTHOR CONTRIBUTIONS

**Seyed Mohammad Seyedmehdi**: Conceptualization; funding acquisition; investigation; resources; visualization; writing—original draft. **Fatemeh Imanparast**: Conceptualization; data curation; funding acquisition; validation; visualization; writing—original draft; writing—review and editing. **Pegah Mohaghegh**: Validation; writing—review and editing. **Saeed Mahmoudian**: Investigation, methodology, project administration, writing—review and editing. **Mona Karimi Dehlaqi**: Investigation, methodology, project administration, writing—review and editing. **Fatemeh Mehvari**: Methodology, project administration, resources, software, writing—review and editing. **Mihan Pourabdollah**: Investigation, methodology, project administration, writing—review and editing.
